# *GDF5* rs143384 Polymorphism Associated with Developmental Dysplasia of the Hip in Brazilian Patients: A Case-Control Study

**DOI:** 10.3390/ijms26115012

**Published:** 2025-05-23

**Authors:** Jamila Alessandra Perini, Raphael Wallace Campos Cunha, Marco Bernardo Cury Fernandes, Lourenço Pinto Peixoto, João Antônio Matheus Guimarães, Amanda dos Santos Cavalcanti, Jéssica Vilarinho Cardoso

**Affiliations:** 1 Research Laboratory of Pharmaceutical Sciences (LAPESF), Rio de Janeiro State University (UERJ), Rio de Janeiro 23070-200, RJ, Brazil; acavalcanti@into.saude.gov.br (A.d.S.C.); jessica_vilarinho@yahoo.com.br (J.V.C.); 2 Hip Surgery Center, National Institute of Traumatology and Orthopaedics (INTO), Rio de Janeiro 20940-070, RJ, Brazil; raphaelwcunha@gmail.com (R.W.C.C.); marcobcury@yahoo.com.br (M.B.C.F.); lourencoppeixoto@gmail.com (L.P.P.); 3 Research Division, INTO, Rio de Janeiro 20940-070, RJ, Brazil; jguimaraes@into.saude.gov.br

**Keywords:** musculoskeletal diseases, *GDF5* gene, genetic polymorphism

## Abstract

Developmental dysplasia of the hip (DDH) is a multifactorial and polygenic abnormal hip joint development, with a prognosis influenced by environmental and genetic factors, potentially leading to complete dislocation. Growth differentiation factor 5 is a signaling molecule, encoded by a polymorphic gene (*GDF5*), promoting the development, repair, and maintenance of bone, cartilage, and other synovial joint tissues. The *GDF5* rs143384 G>A polymorphism affects GDF5 expression and may be associated with a susceptibility to DDH. The aim of this study was to determine the frequency of the *GDF5* rs143384 polymorphism in Brazilian individuals and its influence on the development of DDH. This case–control study included 50 DDH cases and 150 controls without hip disease. Genotyping was performed by real-time PCR using the TaqMan system. The *GDF5* AA variant genotype frequency was significantly higher in DDH cases (32%) compared to controls (14%, *p*-value = 0.01) and showed a marginal association with disease risk (OR = 1.47; CI 95% = 0.96–2.26). The *GDF5* rs143384 polymorphism could be useful in identifying individuals at risk, guiding personalized treatment strategies, and contributing to diagnosis and clinical management.

## 1. Introduction

Developmental dysplasia of the hip (DDH) is the most common congenital skeletal dysplasia, characterized by an abnormal relationship between the femoral head and the acetabulum, with signs of dislocation, subluxation, and femoral head instability [1]. The global prevalence of radiographic DDH in asymptomatic young adults is approximately 2.3% and is more common in women (3.8%) than in men (2.7%) [2], while the incidence ranges from 0.1 to 6.6 per 1000 live births [3], and in Brazil, it ranges from 0.5% to 5%, depending on the region studied [4,5]. DDH after skeletal maturity is a risk factor for early-onset hip osteoarthritis (known as secondary coxarthrosis) [1,6], a pathology characterized by hip cartilage wear, pain, limited movement, and loss of function, which can progress to complete joint dysfunction in advanced stages [7]. DDH has multifactorial etiologies, making it difficult to understand, diagnose, and treat, and significantly impacting patients’ quality of life, leading to impaired social participation, increased social isolation, and a higher economic burden [8,9,10]. To avoid long-term complications, timely diagnosis and intervention are essential [6].

The influence of genetic factors in the development of DDH is widely recognized [11,12]. The gene encoding the protein growth differentiation factor 5 (GDF5), which is involved in the development and maintenance of intra-articular structures, stands out [13]. Reduced expression of *GDF5* may affect acetabular morphology and the development and maintenance of the hip joint ligaments, contributing to the onset and progression of hip disease [14]. Among the polymorphisms described in the *GDF5* gene, the rs143384 G>A SNP, located in the 5′ non-coding region (5′-UTR), stands out for influencing the expression levels of *GDF5*, since the presence of the rs143384A allele reduces its transcriptional activity [15,16,17], in addition to being the most common in different populations https://www.ncbi.nlm.nih.gov/snp/rs143384, (accessed on: 29 March 2025). However, this polymorphism has not yet been analyzed in the Brazilian population. Additionally, the *GDF5* rs14338 SNP has been associated with an increased risk of several musculoskeletal diseases [17,18,19,20,21,22], including dysplasia [11,12,23].

Due to the high frequency of *GDF5* rs143384 in different populations, the functional effect of the SNP on gene expression, and the role of the gene in hip diseases, the present work aimed to describe the frequency of this SNP, and the genetic susceptibility to DDH in the Brazilian population.

## 2. Results

**Figure 1:**
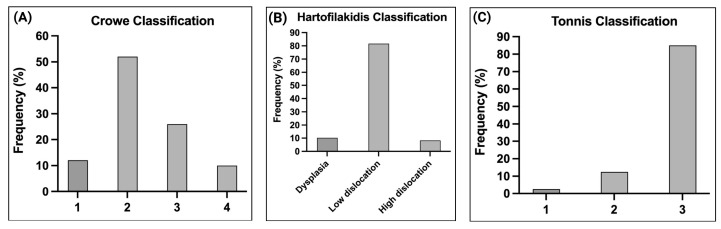
Distribution of DDH cases according to the Crowe, Hartofilakidis, and Tonnis classification. (**A**) The classification of the Crowe from 1 to 4. (**B**) The Hartofilakidis classification: dysplasia, low dislocation, and high dislocation. (**C**) The Tonnis classification from 1 to 3.

The vast majority (78%) of DDH cases had femoral head subluxation (Crowe 2 and 3) (Figure 1A), 82% had low dislocation according to the Hartofilakidis classification (Figure 1B), and 85% had severe osteoarthritis (Tonnis 3) (Figure 1C). Table 1 shows the clinical and functional characteristics of patients diagnosed with DDH. Most individuals have moderate to severe lameness (84%) and moderate to severe pain (82%), with 26.5% having some deformity. A significant number of individuals have difficulty or are unable to put on socks (94%). Half of the patients almost always use a cane for long walks, and the majority need to use a railing or make great effort to get up and down stairs (90%). Approximately 60% can only sit for up to half an hour or cannot sit without pain. Regarding walking distance, about 54% can only walk up to three blocks or around the house, and almost 88% need public transportation. Most of the patients had lived with the disease for more than 21 years (62%), with bilateral involvement.

Table 2 shows the minor allele frequencies (MAF) of the *GDF5* rs143384 “A” in the Brazilian control group compared to global populations.

Table 3 shows the categorical sociodemographic data of the controls and DDH patients included in the case–control study (n = 200). The median age and BMI of DDH patients were 45 years (24–71) and 27.2 kg/m^2^ (16.7–38.4), respectively, while those of controls were 35 years (18–59) and 27.1 kg/m^2^ (17.9–43.3), respectively.

**Figure 2:**
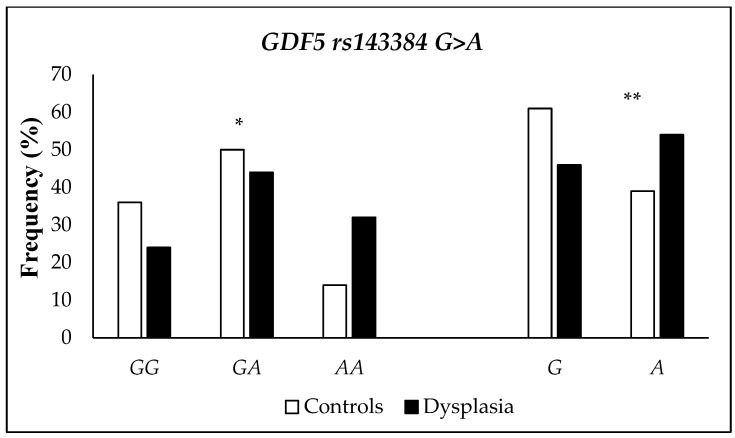
Genotypic (GG, GA, and AA) and allelic (G and A) distribution of the *GDF5* rs143384 polymorphism in the controls (n = 150) and dysplasia cases (n = 50) groups. Pearson’s Chi-square test (X^2^). * *p*-value = 0.01 for genotypes. ** *p*-value = 0.009 for alleles.

The frequency of the *GDF5* rs143384 G>A SNP is in HWE in the studied population (controls and DDH cases; *p*-value = 0.08 and 0.42, respectively). The genotypic and allelic distribution of the *GDF5* rs143384 polymorphism differed between the control and DDH groups, with the variant A allele and the AA genotype being more frequent in cases than in controls (Figure 2). An association analysis was performed to assess the risk of the *GDF5* rs143384 G>A SNP with the susceptibility to DDH disease (Table 4). The *GDF5* rs143384 polymorphism was associated with an approximately 3-fold increased risk of DDH in the presence of the homozygous variant genotype (AA) compared to the wild-type genotype (GG or recessive model GG + GA). After adjusting for sex and age, which remained in the final logistic regression model (*p* < 0.05), and self-reported skin color, there was a borderline association between the homozygous variant genotype (*GDF5* rs143384 AA) and DDH risk in the recessive model. An additive genetic model was also tested by coding the genotypes as continuous variables (GG = 0, GA = 1, AA = 2) in the logistic regression analysis, and a borderline risk was observed between case and control after adjustment for sex and age (OR = 1.54; 95% CI = 0.95–2.57).

## 3. Discussion

The current study describes the association of a polymorphism of a gene (*GDF5*) involved in the pre- and postnatal development of the hip with the risk of DDH in patients from a public orthopedic referral hospital in Brazil. Of the 578 cases treated at the institute for surgical treatment of any hip disease, 8.7% of them had a confirmed diagnosis of DDH. This frequency was higher than that reported in the global [24] and Brazilian [4,5] populations because patients were referred from other institutions for surgical treatment at a highly complex reference center. Thus, in our sample, most DDH patients had the disease for more than 10 years and the age range was above 18 years, suggesting that DDH progresses over time, as has been described in North American and Canadian populations [25].

In terms of functional and mobility characteristics, the majority of DDH cases reported difficulty climbing stairs and putting on socks, highlighting the impact of hip dysplasia on vertical mobility and reflecting limitations in hip flexibility and range of motion. These findings are consistent with the loss of function in activities of daily living (e.g., sitting and walking) and pain reported in the present study, and have also been observed in Australian [26] and Japanese [27] populations. Furthermore, in this study, most DDH cases showed bilateral involvement, which is similar to the frequency found in previous studies (61%) [27,28]. However, the American Academy of Pediatrics states that the left hip is three times more likely to be affected than the right hip in the US population [29]. This variance also suggests a possible genetic predisposition [30] or systemic factors [31] contributing to DDH, which has a complex and multifactorial etiology [32]. Therefore, it is crucial to investigate the causes of DDH, as the disease results in physical and functional limitations for patients and is costly to diagnose and treat [33,34].

Our multidisciplinary team is dedicated to searching for genetic variations associated with susceptibility to musculoskeletal disease, with the aim of monitoring those at risk and thus avoiding frequent, mainly radiological, follow-up examinations, a poor quality of life, and the high costs associated with diagnosing and treating orthopedic disease [35,36,37,38,39]. This is the first study to describe the frequency data of the *GDF5* rs143384 G>A SNP in the Brazilian population and to evaluate its association with hip disease. This SNP is the most common in different populations, ranging from 37% to 81% [11,12,17,19,20,21,23]; in addition, it reduces the expression of GDF5, which is crucial for skeletal formation and joint development [23]. It is not appropriate to extrapolate data from other ethnic groups because the Brazilian population has been formed by extensive admixture from different ancestral roots. Compared to global populations, the Brazilian frequency of the *GDF5* rs143384 A allele was significantly lower than that observed in American, Amerindian, European, and East Asian populations, but significantly higher than that observed in African and African American populations. However, the frequency of the *GDF5* rs143384 A allele was similar to that found in South Asian populations. These findings confirm the genetic diversity and complex admixture patterns of the Brazilian population. In addition, Pena and colleagues, in a multicenter genomic ancestry study conducted in different regions of Brazil, demonstrated that the genetic ancestry of individuals classified as white, black, or other in Brazil is highly heterogeneous because of extensive admixture from the ancestral roots of Amerindians, Europeans, and Africans [40].

In a GWAS study involving 5411 individuals (cases and controls), the *GDF5* rs143384 A allele was associated with DDH, with a 1.44-fold risk [11]. In a systematic review with a meta-analysis of 45 studies including 11,489 cases, the *GDF5* rs143384 SNP showed the most robust association with the DDH phenotype [12]. In this sense, our findings repeat the discussion initiated in 2010 in a study conducted on a European population, which observed the same association between the *GDF5* rs143384 A allele and twice the risk of developing DDH [23]. The presence of the *GDF5* rs143384 A allele leads to a decrease in GDF5 transcriptional activity [16,17], which may explain the association with DDH, as loss of function of the GDF5 protein, which is active during intrauterine life, can lead to musculoskeletal developmental malformations [22].

Despite the advances made by this study, it has some limitations: there was potential for recall bias in the data, particularly regarding conditions related to childbirth and family history of the disease, as it was a retrospective study. Another limitation is the lack of analysis of other genes such as *CX3CR1*, *TENM3*, *TGFB1* and *TXNDC3* [12,13], which may also influence the risk of DDH in our Brazilian cohort. Therefore, future studies evaluating variants in these genes in the etiopathogenesis of DDH in a heterogeneous population, as well as studies that consider individual genetic ancestry data or equivalent measures (e.g., principal components), would be of great interest. This is because the associations found may be because of a population’s genetic structure. However, there are notable points that should be highlighted: (i) all patients were evaluated by experienced orthopedic surgeons specializing in hip conditions, who confirmed the diagnosis and excluded other hip deformities; (ii) the significant number of controls (1:3); (iii) the pioneering effort to evaluate a mixed population such as the Brazilian one, comparing the possible association of the SNP with a relevant hip disease.

This is the first study to reflect the reality of a tertiary public hospital in a developing country involved in the diagnosis and management of complex and multifactorial hip disease, combining research into genetic biomarker identification and clinical/medical care. The data from this study may assist in the identification of individuals at risk of developing DDH, allowing for early diagnosis and avoiding frequent surveillance, excessive radiographs, poor quality of life, and high treatment costs.

## 4. Materials and Methods

### 4.1. Study Population

**Figure 3:**
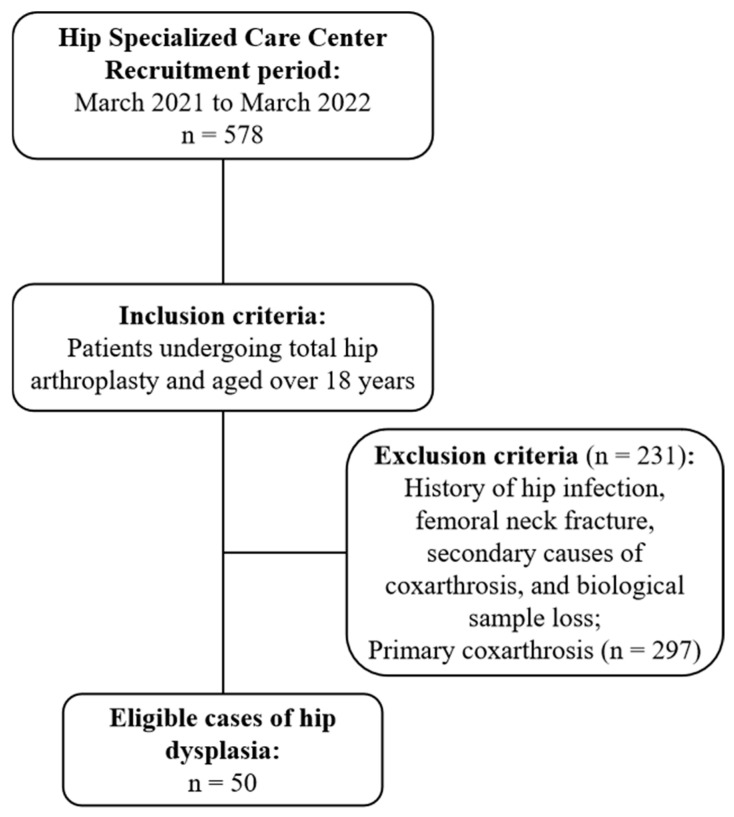
Flowchart of the study participant selection process.

The observational case–control study was approved by the Institutional Review Board (protocol number 40817120.1.0000.5273) and conducted from March 2021 to March 2022. Patients undergoing total hip arthroplasty at the Hip Specialized Care Center of the National Institute of Traumatology and Orthopedics (Rio de Janeiro/Brazil), with a diagnosis of DDH at the time of consultation, aged 18 years or older, were included. The absence of biological material for polymorphism analysis, history of hip infection, femoral neck fracture, and primary and secondary causes of coxarthrosis corresponded to the exclusion criteria. A total of 578 patients were enrolled, and 50 DDH cases remained after the exclusion criteria were applied (Figure 3). All measurements and clinical assessments were performed independently by at least two investigators (R.W.C.C., L.P.P., and/or M.B.C.F.) who are experienced hip surgeons and who were blinded to the clinical information to avoid bias.

The control group consisted of healthy volunteers without hip disease who were recruited from the hospital blood donor bank when they showed up to donate blood (n = 389). The control group was evaluated by an orthopedic hip surgeon to rule out any hip deformity. From this pool, 150 controls were selected using simple random sampling (1:3 ratio) for the case–control study, employing the =RAND() function in Microsoft Excel to ensure an equal probability of selection among all eligible individuals. To ensure that this selection did not introduce bias into the study, we compared the excluded (n = 239) and included (n = 150) control subjects in terms of sex, age, BMI, and self-reported race/color. No significant differences were found between the groups (*p* > 0.05).

All subjects completed a demographic questionnaire and self-identified according to the classification scheme used in the Brazilian census https://censo2022.ibge.gov.br/panorama/ (accessed on 15 February 2025), which is based on self-perception of skin color. Accordingly, the individuals were distributed in two groups [41]: white (n = 124 controls) and non-white (n = 265 controls).

Body mass index (BMI) was classified into five groups according to the World Health Organization [41] and calculated as weight (kg) divided by the square of height (m^2^): underweight (BMI ≤ 18.5 kg/m^2^), normal weight (18.6–24.9 kg/m^2^), overweight (25–29.9 kg/m^2^), obesity (30–39.9 kg/m^2^), and morbid obesity (BMI ≥ 40 kg/m^2^).

### 4.2. Clinical Evaluation

**Figure 4:**
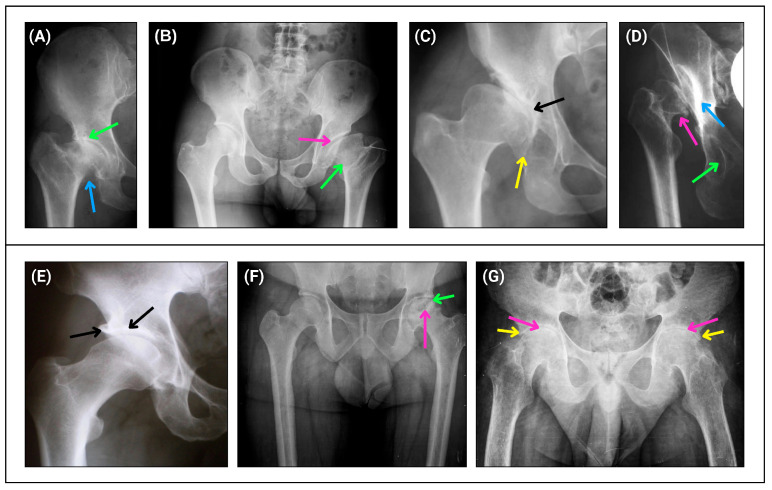
Radiographic representations of the Crowe (**A**–**D**) and Tonnis (**E**–**G**) hip classifications of dysplasia cases. (**A**) The green arrow indicates a reduced joint space with a reduced femoral head in the acetabulum, and the blue arrow indicates a short femoral neck (Crowe 1 and no subluxation). (**B**) The pink arrow indicates a femoral head with loss of acetabular coverage and the green arrow indicates a short femoral neck (Crowe 2 and low dislocation). (**C**) The black arrow indicates a previous acetabular roof with advanced degenerative changes due to abnormal contact between the femoral head and the acetabulum, and the yellow arrow indicates a previous acetabular roof (Crowe 3 and low dislocation). (**D**) The blue arrow indicates a new acetabulum due to abnormal contact between the parts, the green arrow indicates the previous/native acetabulum, and the pink arrow indicates a dislocated and deformed femoral head (Crowe 4 and high dislocation). (**E**) The black arrow on the left indicates an osteophyte and the black arrow on the right indicates a sclerosis of the acetabular roof (Tonnis 1). (**F**) The pink arrow indicates a cyst in the femoral head, and the green arrow shows a moderate reduction in the joint space (Tonnis 2). (**G**) The pink arrow indicates advanced joint space reduction and the yellow arrow indicates an osteophyte in the acetabular roof (Tonnis 3).

DDH was diagnosed radiographically by the presence of one of the following parameters: center–edge angle less than 20º and break in Shenton’s arc. The disease was graded from 1 to 4 according to Crowe’s classification based on the severity of the dysplasia. Grade 1 is characterized by a reduced hip (incongruent but without subluxation) (Figure 4A); grade 2 by less than 50% hip dislocation (low dislocation) (Figure 4B); grade 3 by 50 to 100% hip dislocation (low dislocation) (Figure 4C); and grade 4 by more than 100% hip dislocation (high dislocation) (Figure 4D). The Hartofilakidis classification was also used, categorizing cases as reduced hip (without subluxation), low dislocation, and high dislocation. DDH cases with secondary coxarthrosis (hip osteoarthritis) were also graded according to the Tonnis classification, which includes four progressive grades, including Tonnis angle greater than 10° and Sharp angle greater than 45°. Coxarthrosis was diagnosed by plain pelvic radiography, which showed a reduction in the hip joint space, with a normal value of 3mm. Thus, according to the Tonnis classification, in grade 0, there is no disease; therefore, the joint is normal. Grade 1 represents a mild reduction in the joint space between the acetabulum and the femoral head (Figure 4E). Grade 2 shows a moderate joint space reduction with osteophytes, metaphyseal cysts, and sclerosis (Figure 4F). Grade 3 exhibits a marked reduction in the joint space, with more prominent osteophytes, an increased presence of subchondral cysts, and increased bone sclerosis (Figure 4G).

### 4.3. Sample Collection and Polymorphisms Genotyping Analysis

Oral mucosal epithelium was collected using a swab or blood sample in an EDTA tube for subsequent DNA extraction, using a Qiagen extraction kit (Hilden, Germany) according to the manufacturer’s recommendations. Genotyping analysis of the *GDF5* rs143384 G>A SNP was performed using TaqMan allelic discrimination assays (C____599144_1_, Thermo Fisher Scientific, Carlsbad, CA, USA). Real-time PCR reactions were carried out in a final volume of 8 μL containing 30 ng of DNA, 1× Taqman Universal Master Mix (Applied Biosystems, Foster City, CA, USA), 1× of each specific oligo and probe assay, and H_2_O to a final volume. The PCR conditions were as follows: 95 °C for 10 min followed by 40 cycles of denaturation at 92 °C for 15 s and annealing at 60 °C for 1 min. Allele detection was performed after 1 min at 60 °C on the 7500 Real-Time System (Applied Biosystems). Each genotyping assay used two negative and two positive controls for each genotype (wild-type, heterozygote, and variant homozygote).

### 4.4. Data Analyses

Population normality was assessed using the Shapiro–Wilk test. Continuous variables were expressed as medians with minimum and maximum values, and differences between means were analyzed using Mann–Whitney test. Categorical data were expressed as numbers (n) and frequencies (%) and analyzed using Pearson’s chi-square test (χ2) or Fisher’s exact test, when appropriate.

The allelic and genotypic frequencies of the *GDF5* rs143384 G>A SNP were determined by direct allele counting, followed by the Hardy–Weinberg equilibrium (HWE) calculation. Allele and genotype frequencies between groups (cases and controls) were compared using the χ2 test or Fisher’s exact test, as appropriate. Three genetic models were tested to assess the association between the *GDF5* rs143384 SNP and DDH: genotypic (GG, GA and AA), recessive (AA vs. GA + GG), and additive. For the additive model, genotypes were coded as a continuous variable (GG = 0, GA = 1, AA = 2) in the binary logistic regression analysis, allowing for the evaluation of a dose–response relationship. The magnitude of the association between the presence of the *GDF5* rs143384 SNP and the development of DDH was estimated by odds ratios (ORs) with their respective 95% confidence intervals (CI). Logistic regression models were applied to adjust the risk for potential confounding factors, selected based on statistical significance (sex and age) and biological relevance (self-reported race/color). Statistical analyses were performed using the SPSS statistical package, version 20.0, and a *p*-value ≤ 0.05 was considered statistically significant.

## 5. Conclusions

The results of this study revealed a marginal association between the *GDF5* rs143384 SNP and an elevated risk of DDH. Due to the progressive nature of DDH, identifying this association may provide a genetic basis for early screening and the prevention of complications.

## Figures and Tables

**Table 1:** Clinical and functional characteristics of patients with DDH (n = 50).

Characteristics	n (%)	Characteristics	n (%)	Characteristics	n (%)
*Lameness*		*Support*		*Climbing and descending stairs*	
None	2 (4)	None	22 (44)	Normally	3 (6)
Mild	6 (12)	One crutch	1 (2)	With support on the handrail	29 (58)
Moderate	24 (48)	Cane almost always	20 (40)	With great effort	16 (32)
Severe	18 (36)	Cane for long walks	5 (10)	Cannot	2 (4)
		Missing	2		
*Pain*		*Sit*		*Distance able to walk*	
None	2 (4)	For one hour	13 (26)	Home walker	11 (22)
Mild	5 (10)	For up to one hour	7 (14)	Up to three blocks	16 (32)
Moderate without limit	13 (26)	For up to half an hour	23 (46)	Three blocks	9 (18)
Moderate with limit	13 (26)	Cannot sit without pain	7 (14)	Up to six blocks	6 (12)
Intense	15 (30)			Six blocks	4 (8)
Disabling	2 (4)			Unlimited	4 (8)
*Putting on socks*		*Deformity*		*Public transport*	
No difficulties	3 (6)	No	36 (73.5)	No	6 (12.2)
With difficulties	31 (62)	Yes	13 (26.5)	Yes	43 (87.8)
Cannot	16 (32)	Missing	1	Missing	1
*Disease side*		*Disease evolution*			
Right	10 (20)	<20 years	19 (38)		
Left	16 (32)	21–30 years	24 (48)		
Bilateral	24 (48)	>40 years	7 (14)		

**Table 2:** Global allele distribution of the *GDF5* rs143384 polymorphism.

Cohorts	N ^a^	rs143384 A Allele	*p*-Value ^b^	Reference
Brazilian	778	0.419	Reference	Present study
American	13,356	0.666	<0.001	gnomAD ^c^
African	1396	0.011	<0.001	gnomAD ^c^
African American	74,830	0.104	<0.001	gnomAD ^c^
Amerindigenous	4525	0.873	<0.001	gnomAD ^c^
European	9233	0.555	<0.001	gnomAD ^c^
East Asian	44,794	0.741	<0.001	gnomAD ^c^
South Asian	90,402	0.426	0.69	gnomAD ^c^

^a^ Number of chromosomes (20q11.22) of *GDF5* loci. ^b^ *p*-value from Chi-square test (https://www.icalcu.com/stat/chisqtest.html accessed on: 29 April 2025) was performed using the allele frequencies observed in the Brazilian population of the present study as the reference. ^c^ Data from https://gnomad.broadinstitute.org/variant/20-35437976-G-A?dataset = gnomad_r (accessed on: 29 April 2025).

**Table 3:** Sociodemographic data of controls (n = 150) and developmental hip dysplasia cases (n = 50).

Characteristic	Controls n (%)	DDH n (%)	*p*-Value
*Age (years)*			
≤30	56 (37.3)	7 (14)	<0.001
31–40	44 (29.3)	10 (20)	
41–50	32 (21.3)	13 (26)	
51–60	18 (12.0)	13 (26)	
≥61	0 (0)	7 (14)	
*BMI (kg/m* ^2^ *)*			
≤18.5	1 (0.7)	2 (4)	0.19
18.6–24.9	47 (31.3)	10 (20)	
25–29.9	54 (36)	17 (34)	
30–39.9	48 (32)	21 (42)	
*Sex*			
Men	77 (51.3)	14 (28)	0.004
Women	73 (48.7)	36 (72)	
*Skin color* ^a^			
White	52 (34.7)	28 (56)	0.008
Non-white	98 (65.3)	22 (44)	

DDH: developmental dysplasia of the hip. BMI: body mass index. ^a^ Self-declared skin color.

**Table 4:** Analysis of the association between the *GDF5* rs143384 and the susceptibility to DDH.

Polymorphism rs143384 G>A	Controls (n = 150)	DDH (n = 50)	*p*-Value	OR (95% CI)	*p*-Value	ORa (95% CI)
*Genotypes*	n (%)					
GG	54 (36)	12 (24)		1 ^a^		1 ^a^
GA	75 (50)	22 (44)	0.49	1.32 (0.60–2.90)	0.81	0.90 (0.38–2.12)
AA	21 (14)	16 (32)	**0.006**	**3.43 (1.39–8.45)**	0.25	1.39 (0.79–2.44)
GG + GA	129 (86)	34 (68)	**0.005**	1 ^a^	0.08	1 ^a^
AA	21 (14)	16 (32)		**2.89 (1.36–6.13)**		1.47 (0.96–2.26)

Significant values (*p* < 0.05) are shown in bold. DDH: developmental hip dysplasia. ORa: odds ratio adjusted by sex, age, and self-declared skin color. CI: confidence interval. *p*-value: Pearson’s Chi-square test (X^2^). ^a^ Reference group.

## Data Availability

The data presented in this study are available on request from the corresponding author.
